# Corticosteroids vs autologous blood injection for lateral epicondylitis: Study protocol for a cohort trial

**DOI:** 10.1097/MD.0000000000023842

**Published:** 2020-12-18

**Authors:** Chaodong Zhou, Lu Wang

**Affiliations:** Department of Joint Surgery, Chongqing General Hospital, Chongqing, China.

**Keywords:** autologous blood injection, corticosteroids, lateral epicondylitis, protocol

## Abstract

**Background:**

There is limited evidence to assess the evaluation of the safety and effectiveness of autologous blood injections in the treatment of lateral epicondylitis patients. For this study, the aim was to compare the efficiency of corticosteroid and autologous blood injections for the treatment of lateral epicondylitis in a retrospective cohort trial in our single center.

**Methods:**

After being approved by the institutional review committee of Chongqing General Hospital (IRB# 2018.417.C, November 9, 2018), we performed a single-center, retrospective study between November 2018 and January 2020. All participants provided written informed consent. The criteria for inclusion in our experiment are as follows: over 18 years old; with the history of at least 6 months of lateral epicondylitis; and the palpation of lateral epicondyle tenderness; visual analog scale (≥4). In the group A, the patient were injected the autologous blood. In group B, the patients were immersed with 0.5% of bupivacaine (1 ml) and local corticosteroids (2 ml) at lateral epicondyle. The outcomes were composed of a visual analog scores of subjective pain severity over the past 24 hours as the primary result; and limb function in various tasks of daily activity measured with disabilities of the arm, shoulder, and hand quick questionnaire scores, the maximum grip strength and the modified scores of Nirschl, as secondary results. All the results were assessed before the injection and at 4 weeks and 8 weeks after the injection. For all examination, when the *P* value was less than .05, it would be defined to be a statistically significant difference.

**Results:**

The results of this study would provide new information about the influence of autologous blood injections in treating the lateral epicondylitis.

**Trial registration:**

This study protocol was registered in Research Registry (researchregistry6263).

## 1 Introduction

Among patients reporting elbow pain, lateral epicondylitis is a common disease and the most common diagnosis. It occurs in 4 adults per thousand adults every year, especially in those between 35 and 54 years old.^[1–3]^ The most common symptom is pain on the outside of the elbow, which is exacerbated by the wrist's grip and dorsiflexion against resistance. This disease was first recognized more than 100 years ago and is believed to be caused by the degeneration of familiar extensor tendon.^[4–8]^

For the external epicondylitis, the conservative treatment contain shock wave therapy, eccentric exercise, corticosteroid injections, and splinting.^[9,10]^ The long-term effects of the above treatment schemes are reported to be similar. Nevertheless, some patients are not sensitive to the conservative treatment and they will undergo the chronic pain for more than 6 weeks.^[11]^ Despite the surgery is effective and beneficial for the patients, it is destructive and invasive. At present, autologous blood injections designed according to the underlying pathophysiological mechanism of lateral epicondylitis (tendons degeneration resulted from microvascular interaction and mechanical overload rather than the process of inflammation) have been used as alternative treatment options.^[12–15]^ From the peripheral vein, autologous blood can be taken, and it includes a variety of cell mediators and hormones, which can facilitate tendon cell differentiation, replace the degenerative cells and then promote the healing of tissue. In former studies, autologous blood has been utilized in the treatment of chronic tendon diseases, and satisfactory outcomes have been achieved.^[16–18]^

However, there is limited evidence to assess the evaluation of the safety and effectiveness of autologous blood injections in the treatment of lateral epicondylitis patients. For this study, the aim was to compare the efficiency of corticosteroid and autologous blood injections for the treatment of lateral epicondylitis in a retrospective cohort trial in our single center. The results of this study would provide new information about the influence of autologous blood injections in treating the lateral epicondylitis.

## 2 Materials and methods

### 2.1 Study design

After being approved by the institutional review committee of Chongqing General Hospital (IRB# 2018.417.C, November 9, 2018), we performed a single-center, retrospective study between November 2018 and January 2020. All participants provided written informed consent. The study was also registered with Research Registry (researchregistry6263).

### 2.2 Study population

The criteria for inclusion in our experiment are as follows: over 18 years old; with the history of at least 6 months of lateral epicondylitis; and the palpation of lateral epicondyle tenderness; visual analog scale (≥4). Exclusion criteria was set as following: patients with pregnancy, cervical spondylosis, or rheumatologic disease, or severe systemic illness; local injection of corticosteroids for the treatment of lateral epicondylitis in the past 3 months, physical treatment in the past 3 months, and acetaminophen drugs and nonsteroidal anti-infl ammatory drugs during the past week; previous surgery or elbow dislocation; patients with the radiograph and history about elbow and upper extremity arthritis.

### 2.3 Procedure

When the patient is sitting or lying on his back, the elbow is bent 90 degrees and the palms are downward. Anatomical bone markers were determined. In the aseptic prophylaxis, the needle was inserted along supracondylar crest into the proximal lateral epicondyle and slowly extended into the lower surface of radial extensor carpi brevis during infiltration. The small adhesive sterile dressing is utilized. In the group A, the patient were injected the autologous blood. In accordance with this technique, autogenous blood (2 ml) was collected from contralateral upper limb vein and then injected into lateral epicondyle by mixing 0.5% of bupivacaine (1 ml). By using the identical method, in group B, the patients were immersed with 0.5% of bupivacaine (1 ml) and local corticosteroids (2 ml) (80 mg of methylprednisolone acetate) at lateral epicondyle.

### 2.4 Outcome measures

The baseline demographic data was recorded by research assistant, involving physical activity, age, and sex. In addition, the side of the affected hand and the dominant hand, medical history, duration of symptoms, absenteeism, and its duration were also asked. The outcomes were composed of a visual analog scores of subjective pain severity over the past 24 hours as the primary result; and limb function in various tasks of daily activity measured with disabilities of the arm, shoulder and hand quick questionnaire scores, the maximum grip strength, and the modified scores of Nirschl, as secondary results. All the results were assessed before the injection and at 4 weeks and 8 weeks after the injection (Table 1).

**Table 1:** The postoperative outcomes in the 2 groups.

Outcome	Autologous blood group	Corticosteroids group	*P* value
VAS			
SHQQS			
Maximum grip strength			
Nirschl scores			

### 2.5 Statistical analysis

Through applying SAS (version 9.3 and SAS Enterprise Guide 6.1; SAS Institute), the analyses could be conducted. For the analysis, the type I error probability was *P* < .05. The proportions and medians were applied to express the baseline queue features, and the Wilcoxon rank sum tests for continuous variables and Chi-Squared tests for categorical variables between 2 groups were utilized for the comparison of the baseline queue features. For all examination, when the *P* value was less than .05, it would be defined to be a statistically significant difference.

## 3 Discussion

Lateral epicondylitis, more generally known as the tennis elbow, it is often diagnosed in the patients aged between 35 and 50, accounting for about 1% to 3% of general population. The corticosteroid injection is the most commonly utilized injection treatment, which is widely applied in treating tenopathy due to its convenient use and low cost, nevertheless, its effect in improving function and relieving pain is short-term. In recent years, the injection of autologous blood has been utilized as the alternative therapy. Several researches have demonstrated that the corticosteroid injections are beneficial in short term, while the injections of autologous blood may be a more effective treatment for long-term pain relief. For this experiment, the aim was to compare the efficiency of corticosteroid and autologous blood injections for the treatment of lateral epicondylitis in a retrospective cohort trial in our single center. The results of this study would provide new information about the influence of autologous blood injections in treating the lateral epicondylitis.

## Author contributions

Chaodong Zhou planned the study design and wrote the study protocol. Lu Wang reviewed the study protocol. Chaodong Zhou and Lu Wang will recruit participants and collect data. All of the authors have read, commented on, and contributed to the submitted manuscript.

**Conceptualization:** Chaodong Zhou.

**Data curation:** Chaodong Zhou.

**Formal analysis:** Chaodong Zhou, Lu Wang.

**Funding acquisition:** Lu Wang.

**Investigation:** Chaodong Zhou.

**Methodology:** Chaodong Zhou.

**Project administration:** Lu Wang.

**Resources:** Lu Wang.

**Software:** Chaodong Zhou.

**Supervision:** Lu Wang.

**Writing – original draft:** Chaodong Zhou.

**Writing – review & editing:** Lu Wang.
